# Cytokinin signaling regulates two-stage inflorescence arrest in Arabidopsis

**DOI:** 10.1093/plphys/kiac514

**Published:** 2022-11-04

**Authors:** Catriona H Walker, Alexander Ware, Jan Šimura, Karin Ljung, Zoe Wilson, Tom Bennett

**Affiliations:** School of Biology, Faculty of Biological Sciences, University of Leeds, Leeds, UK; School of Biosciences, University of Nottingham, Loughborough, UK; Department of Forest Genetics and Plant Physiology, Umeå Plant Science Centre, Swedish University of Agricultural Sciences, Umeå, Sweden; Department of Forest Genetics and Plant Physiology, Umeå Plant Science Centre, Swedish University of Agricultural Sciences, Umeå, Sweden; School of Biosciences, University of Nottingham, Loughborough, UK; School of Biology, Faculty of Biological Sciences, University of Leeds, Leeds, UK

## Abstract

To maximize reproductive success, flowering plants must correctly time entry and exit from the reproductive phase. While much is known about mechanisms that regulate initiation of flowering, end-of-flowering remains largely uncharacterized. End-of-flowering in Arabidopsis (*Arabidopsis thaliana*) consists of quasi-synchronous arrest of inflorescences, but it is unclear how arrest is correctly timed with respect to environmental stimuli and reproductive success. Here, we showed that Arabidopsis inflorescence arrest is a complex developmental phenomenon, which includes the arrest of the inflorescence meristem (IM), coupled with a separable “floral arrest” of all unopened floral primordia; these events occur well before visible inflorescence arrest. We showed that global inflorescence removal delays both IM and floral arrest, but that local fruit removal only delays floral arrest, emphasizing their separability. We tested whether cytokinin regulates inflorescence arrest, and found that cytokinin signaling dynamics mirror IM activity, while cytokinin treatment can delay both IM and floral arrest. We further showed that gain-of-function cytokinin receptor mutants can delay IM and floral arrest; conversely, loss-of-function mutants prevented the extension of flowering in response to inflorescence removal. Collectively, our data suggest that the dilution of cytokinin among an increasing number of sink organs leads to end-of-flowering in Arabidopsis by triggering IM and floral arrest.

## Teaser

The phytohormone cytokinin regulates multiple distinct developmental events at the end of flowering in *Arabidopsis thaliana.*

## Introduction

In order to maximize reproductive success, flowering plants must simultaneously fulfill three distinct requirements. First, the quantity of reproductive structures produced by the plant—inflorescences, flowers, fruits, and seeds—must be carefully matched to the availability of resources (light, fixed carbon, nitrate, phosphate, and water); both those already acquired by the plant, and those it might yet acquire (Walker and Bennett, 2018). Second, the timing of both the start and end of the reproductive phase must be optimized to occur in the correct season and to coincide with the availability of both pollinators and crucially, potential mates. Third, plants must measure their own reproductive success, and use this information to modify both the quantity of reproductive structures they produce and the timing of their reproductive phase (Walker and Bennett, 2018). Plants typically meet all these criteria, producing a coherent and flexible “reproductive architecture” that can react to changes in circumstance (Walker et al., 2021), but our mechanistic understanding of reproductive architecture control is still limited.

Given our knowledge of shoot branching control in flowering plants, it is very likely that the integration of long-distance hormonal signals plays a key role in determining the quantity of reproductive structures produced. For instance, soil nitrate and phosphate availability respectively upregulate cytokinin synthesis and downregulate strigolactone synthesis in the root (Takei et al., 2001; Umehara et al., 2010). Cytokinin and strigolactones are transported root-to-shoot and are perceived in axillary buds to determine their outgrowth, respectively promoting and repressing outgrowth (reviewed in Wheeldon and Bennett, 2021), and thus connecting the quantity of branches to soil resources. Apical dominance, which is driven by export of the hormone auxin from actively growing shoot apices, also plays a key role in shoot branching regulation by inhibiting the activation of additional axillary buds through the self-organizing properties of the auxin transport system (Prusinkiewicz et al., 2009; Shinohara et al., 2013; Bennett et al., 2016; Van Rongen et al., 2019). Removing actively growing shoots removes this inhibition, and allows new axillary buds to activate and accurately replace the lost branches (Walker et al., 2021); apical dominance thus acts as a mechanism by which plants can “measure” their shoot branching. There is certainly evidence that both fruit and seeds can also act as sources of “dominance” within the reproductive system (Bangerth, 1989), and can prevent new fruit, seed, and inflorescences from developing (Walker and Bennett, 2018; Walker et al., 2021), probably also through their export of auxin (Bangerth, 1989; Lenser et al., 2018; Ware et al., 2020; Haim et al., 2021; Goetz et al., 2021). Furthermore, cytokinin has been shown to mediate the connection between soil nitrate resources and the activity of inflorescence meristems, which initiate new floral meristems (FMs) at a greater rate (“florochron”) as nitrate levels increase (Landrein et al., 2018).

The timing of reproduction—or at least its initiation—is generally very well understood in flowering plants. At least seven distinct environmental or internal cues are integrated together to regulate the floral transition that begins the reproductive phase (Cho et al., 2016; Gol et al., 2017). However, the events that contribute to end-of-flowering are generally much less studied, in part because end-of-flowering is a much more diverse phenomenon than floral transition (González-Suárez et al., 2020). While the floral transition is a single process, there are at least four different developmental processes by which end-of-flowering can occur, and different species use them in different combinations to end their reproductive phase (González-Suárez et al., 2020). For instance, in Arabidopsis (*Arabidopsis thaliana*), end-of-flowering occurs because plants cease to initiate new inflorescences early in flowering and because each inflorescence has a finite developmental lifetime (Ware et al., 2020). End-of-flowering in Arabidopsis was initially proposed to be a synchronized “global proliferative arrest” (Hensel et al., 1994), but recent work demonstrates that each inflorescence stops opening new flowers through a locally mediated process (“inflorescence arrest”) that occurs independently of other inflorescences (Ware et al., 2020). The quasi-synchronous nature of inflorescence arrest in Arabidopsis is mostly explained by the quasi-synchronous initiation of inflorescences (Ware et al., 2020). The timing of inflorescence arrest can be modified by both local and systemic feedback from fertile fruit and inflorescences, forming a flexible system in which developmental timing and measurement of reproductive success are coupled (Hensel et al., 1994; Wuest et al., 2016; Ware et al., 2020).

Most studies have viewed inflorescence arrest as resulting from the arrest of the inflorescence meristem (IM) itself (Hensel et al., 1994; Wuest et al., 2016; Balanzà et al., 2018; Merelo et al., 2022). Certainly, the IM decreases in size and mitotic activity over the course of flowering (Wang et al., 2020; Merelo et al., 2022), before undergoing a regulated arrest toward end-of-flowering (Balanzà et al., 2018; Merelo et al., 2022), entering a quiescent “dormancy-like” state (Wuest et al., 2016) and then undergoing a gradual senescence (Wang et al., 2020). It is also the case that extending the activity of the IM through genetic manipulations in key regulatory genes such as *FRUITFULL* can delay overall inflorescence arrest (Balanzà et al., 2018; Martínez-Fernández et al., 2020; Merelo et al., 2022). However, it is unclear whether the normal end of flower opening in inflorescences is directly caused by IM arrest. Certainly, the FMs in Arabidopsis can also undergo their own arrest (Lenhard et al., 2001; Lohmann et al., 2001; reviewed in Xu et al., 2019), and visible inflorescence arrest could be a result of this process, rather than directly due to IM arrest. Hensel et al. (1994) showed that male sterility and inflorescence/fruit removal (both before and after inflorescence arrest) could extend the lifetime of inflorescence development, either by delaying inflorescence arrest or undoing arrest if it had already occurred. However, it is unclear how the changes in inflorescence arrest in these treatments are actually mediated at a developmental level. In our previous work, we showed that auxin exported from fertile fruits is required for timely inflorescence arrest (Ware et al., 2020), but again, did not identify which tissue is responding to this signal. In the present study, we therefore aimed to define the developmental processes underlying inflorescence arrest in Arabidopsis and to understand in particular the mechanisms by which local and systemic measurement of reproductive success is integrated into these developmental processes.

## Results

### Arabidopsis inflorescence arrest consists of separate IM and floral arrest events

**Figure 1:**
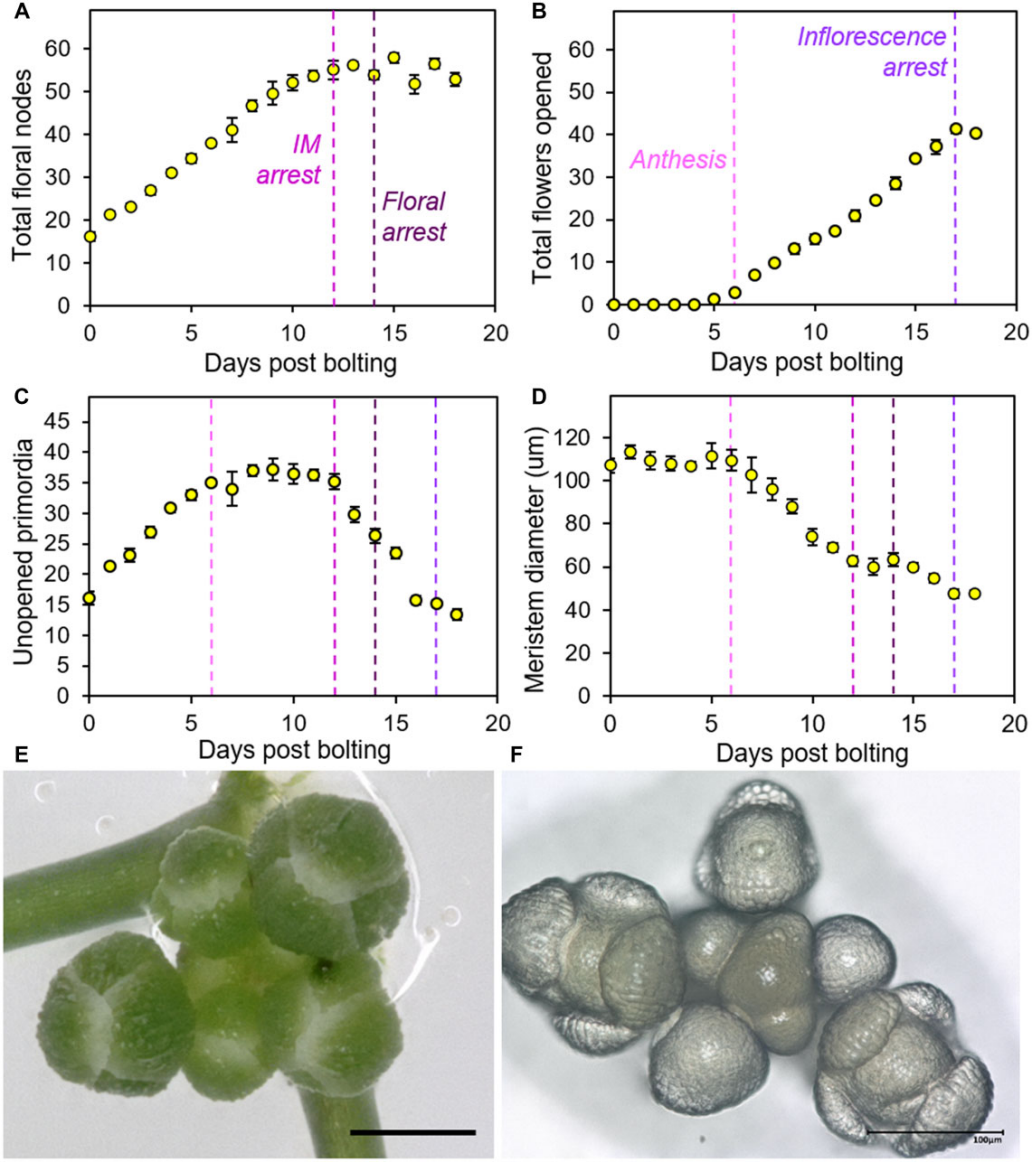
Inflorescence arrest is a two-stage process. Plants were grown in a controlled environment chamber and assigned randomized collection dates. Samples were collected daily from the PI from 0 dpb onwards. A–C, Scatter graphs showing number of floral nodes (A), the number of opened flowers (including previously opened flowers) (B), and the number of unopened floral buds and primordia (C) present along the PI on each day post bolting. D, Scatter graph showing mean IM diameter on each day post bolting. *n* = 4–7 plants dissected each dpb. Error bars indicate sem. The dashed vertical lines indicate the key points in inflorescence lifetime highlighted by this analysis: anthesis (6 dpb), IM arrest (12 dpb), floral arrest (14 dpb), and inflorescence arrest (17 dpb). E, Image showing a typical example of floral buds present within the bud cluster following the final flower opening. Scale bar = 500 µm. F, Image showing IM and remaining attached floral primordia. The meristem is in the center of the bud cluster, with progressively older floral primordia spiralling outward. Scale bar = 100 µm.

To define how Arabidopsis inflorescences arrest, we grew a large population of wild-type (WT) Col-0 plants. Each plant was sampled at a given time point after visible floral transition (days post bolting, dpb) and was destructively analyzed to determine: (1) the number of opened flowers; (2) the number of as-yet-unopened floral primordia and buds; and (3) the total number of floral nodes (i.e. the sum of 1 and 2) on the primary inflorescence (PI) at each time point. In this experiment, we observed that flower opening is a strongly linear process, with plants opening approximately three flowers/day from 6 dpb (i.e. anthesis) until 17 dpb (Figure 1B), at which point the inflorescence arrests. We found that the initiation of floral nodes proceeds at the same linear rate, indicating that flowers mature at a constant rate after initiation (Figure 1A). At the 0 dpb time point, we found that inflorescences had already formed ∼18 primordia, suggesting floral transition actually occurred 6 days before visible bolting. The initiation of floral nodes continued at 3 per day until it plateaued at 12 dpb (Figure 1A). This demonstrates that the IM stops initiating new floral primordia 5 days before visible inflorescence arrest and that in the final phase, the inflorescence is only opening existing floral buds, and not initiating new ones. Our data thus indicate that Col-0 inflorescence lifetime consists of two overlapping stages; an IM-driven flower initiation phase which ends in IM arrest, and an independent flower-opening phase that ends in a “floral arrest” event (Figure 1, A and B).

Consistent with these data, the number of as-yet-unopened floral primordia initially increases until 6 dpb, at which point it plateaus; thereafter, new initiation of primordia is balanced by opening of flowers (Figure 1C). The number of primordia then begins to decline from 12 dpb, since no new floral primordia are being initiated, but flowers continue to open. Primordia number then plateaus again at 17 dpb, after the opening of the final flowers, and the inflorescence arrests with a cluster of ∼15 unopened buds/primordia (Figure 1, C and E). Thus, the final set of floral primordia initiated from 8 to 12 dpb does not open, and the timing of IM arrest does not determine the timing of visible inflorescence arrest.

We also examined the morphology of the IM during this time course. We found that distinct changes in meristem size coincide with changes in flowering (Figure 1D). Interestingly, IM diameter is constant until ∼6 dpb (i.e. anthesis) and then showed two distinct stages of decline in diameter, with the first occurring between 7 and 12 dpb, until the point of IM arrest. After IM arrest, there is a short plateau before a second decline between 15 and 17 dpb, until the point of inflorescence arrest. Thus, physical changes in the IM mirror the discrete stages of inflorescence arrest we have identified. Our results are consistent with recent work, which shows the same decline in IM size over inflorescence lifetime (Wang et al., 2020; Merelo et al., 2022), but provide a more high-resolution time-sequence and more nuanced results.

### Floral arrest is a complex, nonmeristematic phenomenon

**Figure 2:**
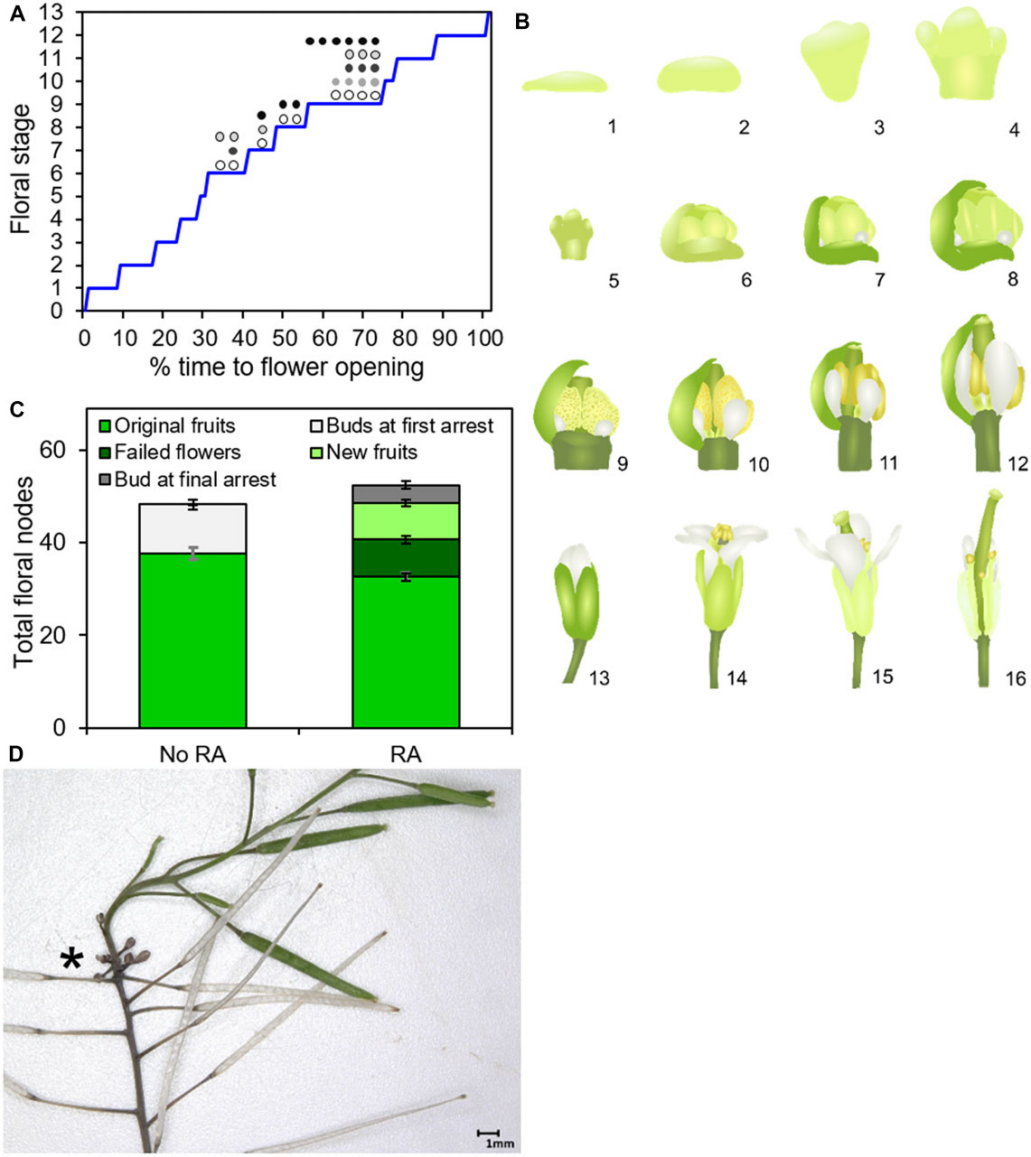
Floral arrest is a complex developmental phenomenon. A, Line graph showing the timing of different floral development stages in Arabidopsis up to flower opening (Stage 13), in relative developmental time (Smyth et al., 1990; see (B) for illustrations). Superimposed are the developmental positions of the oldest floral primordia in the arrested bud clusters of five plants (each circle represents one primordium). B, Cartoon illustrating the floral development stages in Arabidopsis (Smyth et al., 1990). Stages 6–12 are shown in cutaway view, without the enclosing sepals. Key stages for this work are 5 (petal and stamen primordia arise), 6 (sepals enclose bud), 7 (long stamen primordia stalked at base), 8 (locules appear in long stamens), 9 (petal primordia stalked at base), and 10 (petals level with short stamens). C, Stacked bar graph, showing the number of floral nodes on the PI produced in Col-0 plants left untreated for sufficient time, which either reactivated (RA) or did not (no RA). The total floral nodes (i.e. the height of the full stack) are broken down into fruit produced on the PI during initial flowering (mid-green, lower bars), plus either (a) the number of buds and primordia remaining in the bud cluster at first arrest (light gray; untreated plants only) or (b) the number of failed flowers (dark green), new fertile fruits (light green), and the number of buds and primordia remaining in the bud cluster at final arrest (dark gray; treated plants only). Error bars indicate sem, *n* = 21 (no RA), 20 (RA). D, Photo showing within-inflorescence reflowering in Col-0, with older fruit dehisced, a small cluster of characteristic failed flowers (asterisk), and then resumption of a fertile flower opening.

A surprising outcome of our time-course data is the observation that the last flower to open at 17 dpb was initiated at 7 dpb (based on node number), just after anthesis and ∼5 days *before* IM arrest (Figure 1). Thus, all subsequently initiated flowers do not normally open, giving rise instead to the distinctive bud-cluster. Arabidopsis flowers develop and mature in a characteristic and predictable sequence, and thus the stage of development of a given flower reveals its approximate age (Smyth et al., 1990). While Smyth et al. (1990) found that flowers took 13 days to open, flowers only took 10 days to mature under our growth conditions, implying each stage of development occurred at a faster rate. We reasoned that by examining the stage of development of the unopened buds, we could establish approximately when the floral arrest occurs, and we therefore dissected the oldest six to nine floral primordia from arrested bud clusters. We found that the oldest primordia in the cluster are at Stage 9 (*petal primordia stalked at base*), with the stage of development reducing as we moved to progressively younger primordia, consistent with the timeline of Smyth et al. (1990; Figure 2A). Based on these data, it appears that when floral arrest occurs, all buds at Stage 9 or younger halt at their current developmental stage. Stage 9 occurs at ∼70% of the flower development time course (Smyth et al., 1990), which in our conditions would be about 7 days after initiation. Given that the oldest flower in the cluster must have initiated at 7–8 dpb, this places the moment of floral arrest at 14–15 dpb in the above experiment.

An interesting ramification of these data is that flowers older than Stage 9 are “immune” to floral arrest, and go on to fully develop and open. Thus, the final phase of flowering from 15 to 17 dbp consists of the maturation of flowers that were at Stages 10–12 when floral arrest occurred, but which were not “frozen” at that development stage. The partial developmental stasis that results from floral arrest is unanticipated and difficult to explain. It does not seem to reflect changes to the activity of the FM, since FM activity ceases early in flower development once the stamen and carpel primordia form (Stages 5 and 6; Xu et al., 2019). Thus, the arrest of flowers at Stages 6–9 cannot be explained by arrest of the FM.

### Floral arrest is partially reversible, with Stages 5 and 9 as developmental checkpoints

We observed that, if left undisturbed and continually watered after inflorescence arrest, up to 50% of Col-0 plants will naturally re-initiate flower opening on the PI (after a delay of 5–10 days). These tend to be plants that opened a smaller proportion of their flowers in the first place, and may therefore still have available resources to produce more fruit (Figure 2B). These newly opened flowers are always preceded by a run of six to nine “failed flowers” produced by the oldest primordia in the bud cluster (Figure 2D). These were also observed after inflorescence re-activation in the classic study of Hensel et al. (1994). To gain more insight into this process, we dissected the flowers produced during inflorescence re-activation. We observed that all the “failed flowers” were uniformly at Stage 9 of development, with subsequent flowers being in a normal range from Stage 17 downward (Figure 2C). Given that when the floral arrest occurs, primordia are halted in their current stage, these data imply that upon inflorescence re-activation, the oldest six to nine primordia recommence development, but become “stuck” at Stage 9. However, the younger primordia are able to complete development successfully. Based on the number of failed flowers after re-opening, and on our staging of bud clusters (Figure 2A), the failed flowers very likely correspond to the primordia that were at Stages 6–9 when the floral arrest occurred, with flowers at Stage 5 and below being able to form normal, fertile flowers.

These data suggest the unexpected existence of two distinct developmental checkpoints during flower development at Stages 9 and 5. Flowers above Stage 10 seem to be irreversibly committed to opening, but any flower below Stage 9 can be halted in development. Flowers below Stage 5 can successfully re-initiate complete development, but flowers that have passed Stage 5 can only re-initiate development as far as Stage 9, before becoming ‘stuck’. Given that the FM arrests shortly after Stage 5, it is likely that the Stage 5 checkpoint relates to the ability to re-initiate FM activity, but the Stage 9 checkpoint lacks a clear explanation.

### Cytokinin signaling regulates inflorescence arrest

We previously showed that auxin export from fruit formed late in flowering is required for inflorescence arrest (Ware et al., 2020); given the data presented here, we are therefore confident that this auxin export is a key regulator of floral arrest. However, IM arrest occurs too early to be caused by late-formed fruit, and we have previously shown that early-formed fruit have no impact on inflorescence arrest (Ware et al., 2020). It therefore appears unlikely that auxin dynamics regulate IM arrest. Cytokinin is an important root–shoot signal, the availability of which has previously been shown to regulate IM activation and activity in relation to environmental stimuli (Müller et al., 2015; Landrein et al., 2018). We therefore reasoned that IM arrest might be regulated by cytokinin dynamics in the shoot system.

**Figure 3:**
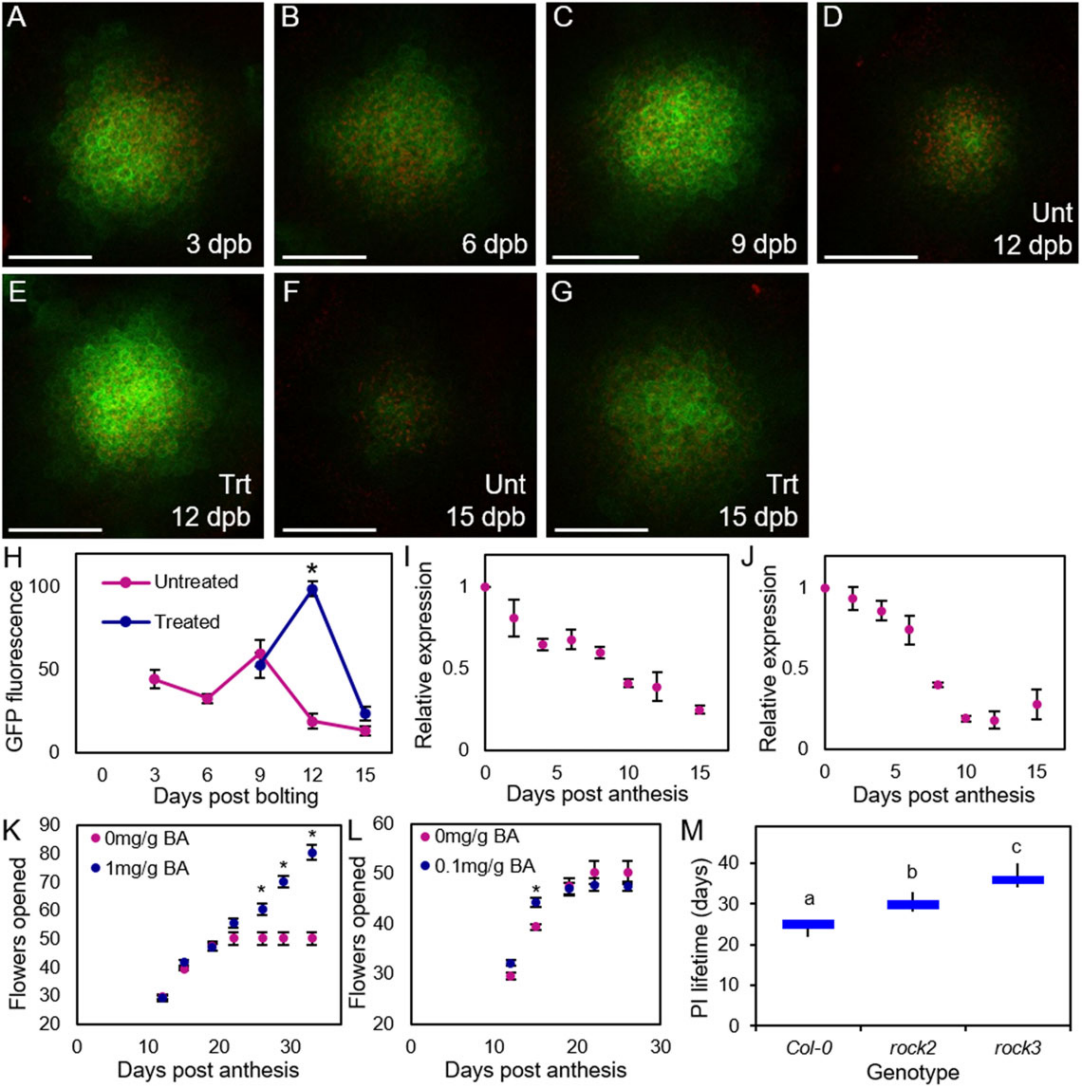
Cytokinin signaling regulates IM arrest. A–G, Confocal microscopy images of primary IMs in Arabidopsis *TCSn:GFP* plants. GFP fluorescence is shown in green and chloroplast autofluorescence in red. Images taken from IMs dissected at 3 (A), 6 (B), 9 (C), 12 (D and E), 15 (F and G) dpb. Plants were either untreated (A–D and F) or treated with the removal of all secondary inflorescences at 6 dpb (E and G). Scale bars = 50 µm. H, Quantification of relative GFP fluorescence (in arbitrary units) in primary IMs of Arabidopsis *TCSn:GFP* plants between 3 and 15 dpb, in untreated plants, or plants treated with removal of all secondary inflorescences at 6 dpb. Data are means of *n* = 5–6 meristems (except 9 dpb treated, *n* = 2) and error bars show sem. Asterisks indicate significant differences in treated samples from untreated control (*t* test, *n* = 5–6, *P* <0.005); other time points are not significantly different. I and J, Relative expression of *ARR5* (I) and *ARR7* (J) in inflorescence apices at different days post anthesis. Quantification of the relative abundance of the transcript of *ARR5* and *ARR7* in inflorescence apices (all unopened buds) in wild-type Col-0 plants harvested following the anthesis of the first flower (Day 0) until inflorescence arrest (Day 15) by RT–qPCR. Data are means of four biological replicates, error bars show sem. K and L, Effect of cytokinin application to fruits on the PI on the duration of flowering, as measured by the rate of fruit production. Fertile L*er* plants were treated from 12-day post anthesis with 6-benzlyaminopurine (BA) dissolved in lanolin treatment at 1 mg·g^−1^ (K), 0.1 mg·g^−1^ (L), or a mock treatment of lanolin only. Error bars show sem. Significant differences between treatments at the same time point are indicated by asterisks (Sidak’s multiple comparisons, on a mixed-effects model, *P* <0.05, *n* = 8 [mock], 9 [0.1 mg·g^−1^], 7 [1 mg·g^−1^]). All other time points were not significantly different between treated and control groups. M, Box plot showing PI lifetime (days) of Arabidopsis cytokinin mutants. Bars with the same letter are not significantly different from each other (ANOVA, Tukey honestly significant difference (HSD) test, *n* = 4–12). Box indicates the interquartile range, internal line shows the median. Whiskers indicate maximum and minimum values.

To test this idea, we first examined cytokinin signaling dynamics in the IM by confocal microscopy, using the *TCSn:GFP* reporter line (Liu and Müller, 2017) to visualize the magnitude of cytokinin signaling over the course of IM lifetime. In untreated plants, we saw a marked decrease in cytokinin signaling in the IM between 3 dpb and 15 dpb, the time frame in which the IM typically arrests (Figure 3, A–H). Consistent with this, using reverse transcription–quantitative polymerase chain reaction (RT–qPCR) we also observed concomitant reductions in the expression of *ARR5* and *ARR7*, two primary cytokinin response genes, in inflorescence apices over the same time frame (Figure 3, I and J). Thus, changes in the IM activity are closely mirrored by changes in CK signaling in the IM.

We next tested whether cytokinin treatment is sufficient to delay inflorescence arrest. We applied 1 mg·g^−1^ cytokinin in lanolin to specific siliques at 12-day post anthesis. We observed a clear delay of inflorescence arrest, with treated plants continuing to produce and open flowers long after control plants had ceased to do so (Figure 3K). The application of 0.1 mg·g^−1^ CK, however, had no obvious effect, with inflorescence arrest and fruit number being the same as untreated plants, showing the effect is strongly dose dependent on cytokinin concentration (Figure 3L). Cytokinin at sufficiently high levels is, therefore, able to extend flowering duration.

We then tested whether mutants with altered cytokinin signaling showed altered inflorescence arrest. We were particularly interested in the *rock2* and *rock3* mutants, which have increased cytokinin sensitivity, and have previously been described as producing more fruit along the main inflorescence before arrest; however, it was not entirely clear whether this was due to increased rate of development or delayed arrest (Bartrina et al., 2017). We observed that *rock2* arrested ∼5 days later than the WT under our conditions, while *rock3* arrested an additional 5 days later than *rock2* (Figure 3M). Taken together, our results therefore strongly suggest that cytokinin regulates the duration of inflorescence activity.

### Cytokinin signaling adjusts both IM and floral arrest

Our results indicated cytokinin was likely a very important factor in regulating inflorescence arrest but did not indicate exactly where cytokinin acts. To understand this, we carefully examined the arrest phenotype in *rock2*, *rock3*, and *ahk2-2 ahk3-3* mutants. The mutants *rock2* and *rock3* have gain-of-function mutations in the cytokinin receptors ARABIDOPSIS HISTIDINE KINASE2 (AHK2) and AHK3, respectively, which confer increased cytokinin sensitivity (Bartrina et al., 2017); the *ahk2 ahk3* double mutant has a loss of function in both receptors, resulting in reduced cytokinin sensitivity (Nishimura et al., 2004; Higuchi et al., 2004).

**Figure 4:**
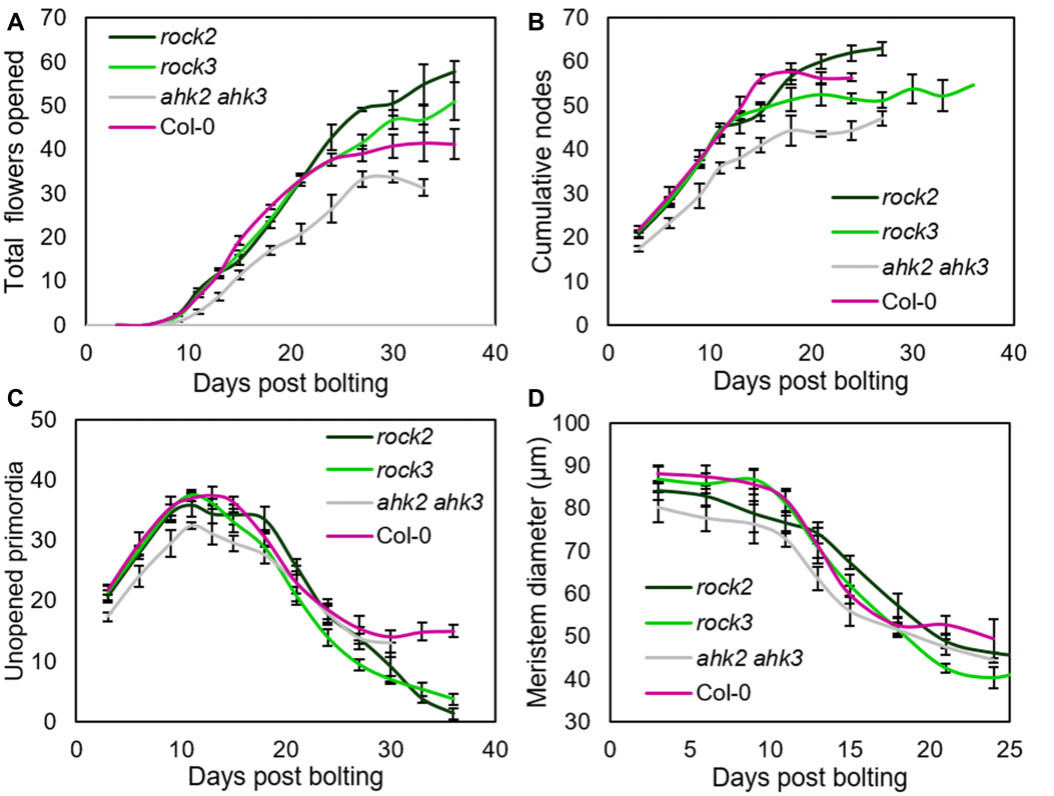
Cytokinin signaling regulates IM and floral arrest. A–D, Large populations of Col-0, *ahk2-2 ahk3-3* (*ahk2/3*), *rock2*, and *rock3* plants were grown under controlled conditions. The timing of visible bolting was recorded for each plant. Plants were randomly assigned to be sampled on a given number of days post bolting, and then destructively sampled at that time point. Time points were spaced every 2–3 days, and 3–12 plants were sampled for each time point. Error bars for all graphs show standard error of the mean. The data presented in Figure 4, A–D are two-time point rolling averages of the raw data presented in Supplemental Figure S2, A–D respectively, in order to show slightly smoothed versions of the data, illustrating the overall trend. A, Scatter graph showing mean opened flowers, at each time point from 0 to 33/36 dpb for each genotype. B, Scatter graph showing the number of total floral nodes present at each time point from 0 to 24/27/36 dpb for each genotype. C, Scatter graph showing the number of unopened primordia present in the inflorescence apex at each time point from 0 to 30/36 dpb for each genotype. D, Scatter graph showing mean IM diameter at each time point from 0 to 24 dpb for each genotype.

As in our earlier experiment (Figure 1), we tracked the number of floral nodes initiated, the number of opened flowers, and the number of unopened buds and primordia on the PI for each genotype over the course of inflorescence lifetime. Control Col-0 plants in this experiment underwent anthesis at ∼7 dpb (Figure 4A), IM arrest at ∼15 dbp (Figure 4, B and C), and inflorescence arrest at ∼24 dpb (Figure 4, A and C). IM diameter decreased between anthesis and IM arrest, as also previously observed (Figure 4D).

We found that *ahk2-2 ahk3-3* (hereafter *ahk2/3*) mutants behave in a similar manner to Col-0 in terms of inflorescence lifetime, undergoing anthesis at ∼7 dpb, IM arrest at ∼15 dpb, and inflorescence arrest at ∼24 dpb. The major effect of *ahk2/3* was a reduction in the rate of IM activity, with fewer nodes initiated each day, leading to fewer flowers opening per day (2.3 versus 1.8 per day in Col-0 and *ahk23*, respectively), and ultimately less nodes and flowers being formed (Day 24: ANOVA + Dunnett’s, *P* < 0.05, *n* = 4). This is highly consistent with previous data showing that cytokinin controls the activity of the IM in response to environmental conditions (Landrein et al., 2018).

The PIs of *rock3* behaved very similarly to Col-0 until inflorescence arrest, although they likely underwent slightly earlier IM arrest than Col-0 (Figure 4, B and C) producing less floral nodes in total (Day 24: ANOVA + Dunnett’s, *P* < 0.05, *n* = 4–5). However, *rock3* plants continued opening flowers for longer than Col-0, until the bud cluster was almost extinct (Figure 4C), opening ∼10 more flowers in total (Figure 4A). The phenotype of *rock3* therefore clearly decouples the two stages of inflorescence arrest; there is no increase in IM activity, but a clear increase in flower opening. We also observed that 30% of IMs in *rock3* also terminated in a terminal flower/fruit, a phenotype never observed in untreated Col-0 (Supplemental Figure S1, A and B).

The PIs of *rock2* also behaved very similarly to Col-0 for the first 10 days of the experiment (Figure 4, A–C), at which point the rate of IM activity seemed to slow down slightly compared to Col-0, (Figure 4B). However, they continued to initiate new floral nodes for longer than Col-0, with IM arrest delayed until ∼22 dpb (Figure 4, B and C), and eventually produced significantly more floral nodes than Col-0 (Day 24: ANOVA + Dunnett’s, *P* < 0.05, *n* = 3–5; Figure 4B). Furthermore, *rock2* mutants also continued opening flowers for longer than Col-0, even taking into account the delay in IM arrest (Figure 4, A and C). They open flowers for ∼14 days after IM arrest, compared to ∼9 days in Col-0, until the bud cluster was almost extinct. Overall, the phenotype of *rock2* mutants is, therefore, qualitatively different from the effect of *rock3*; there is both a delay in IM arrest, with more floral nodes initiated in total, and a subsequent additive delay in floral arrest, with a greater proportion of flowers ultimately opened. Flowers opened until extinction in *rock2*, and we again observed that 30% of IMs in *rock2* terminated in a terminal flower/fruit.

The phenotype of *rock2* and *rock3* indicate that cytokinin might not only control the rate of activity in the IM (Landrein et al., 2018), but also the timing of both IM and floral arrest in inflorescences. The phenotypes of *rock2* and *rock3* are highly consistent with the expression patterns of *AHK2* and *AHK3*. *AHK2* is strongly expressed in both IMs and flowers, and *rock2* affects the arrest of both IMs and flowers; *AHK3* is primarily expressed in flowers, and *rock3* primarily affects the arrest of flowers

### Global inflorescence removal prolongs IM activity; local fruit removal prolongs flower opening

Collectively, our data (Figures 1 and 2) show that visible inflorescence arrest is an unexpectedly complex phenomenon that occurs as a result of separate IM arrest and floral arrest events that occur prior to visible inflorescence arrest. Our data also indicate that these events are separable since *rock2* and *rock3* mutants affect these processes differentially. However, our data do not establish the functional relevance of these different events. The phenotypes of *rock2* and *rock3* mutants are qualitatively similar to those described by Hensel et al. (1994) in both male sterile mutants and in plants treated by inflorescence or fruit removal. We thus hypothesized that floral arrest and IM arrest are separable processes that allow plants to flexibly and homeostatically respond to changes in the plant’s reproductive success either locally (on the same inflorescence) or globally (on all inflorescences). To test this idea, we performed different treatments on Col-0 plants that, based on previous reports, we hypothesized would delay the timing of either IM arrest or floral arrest on the PI. Firstly, we continuously removed all inflorescences except the PI from plants 6 dpb onwards, prior to IM arrest (Hensel et al., 1994), and secondly, we continuously removed fruit from the PI alone from 14 dpb, prior to visible inflorescence arrest (Ware et al., 2020).

**Figure 5:**
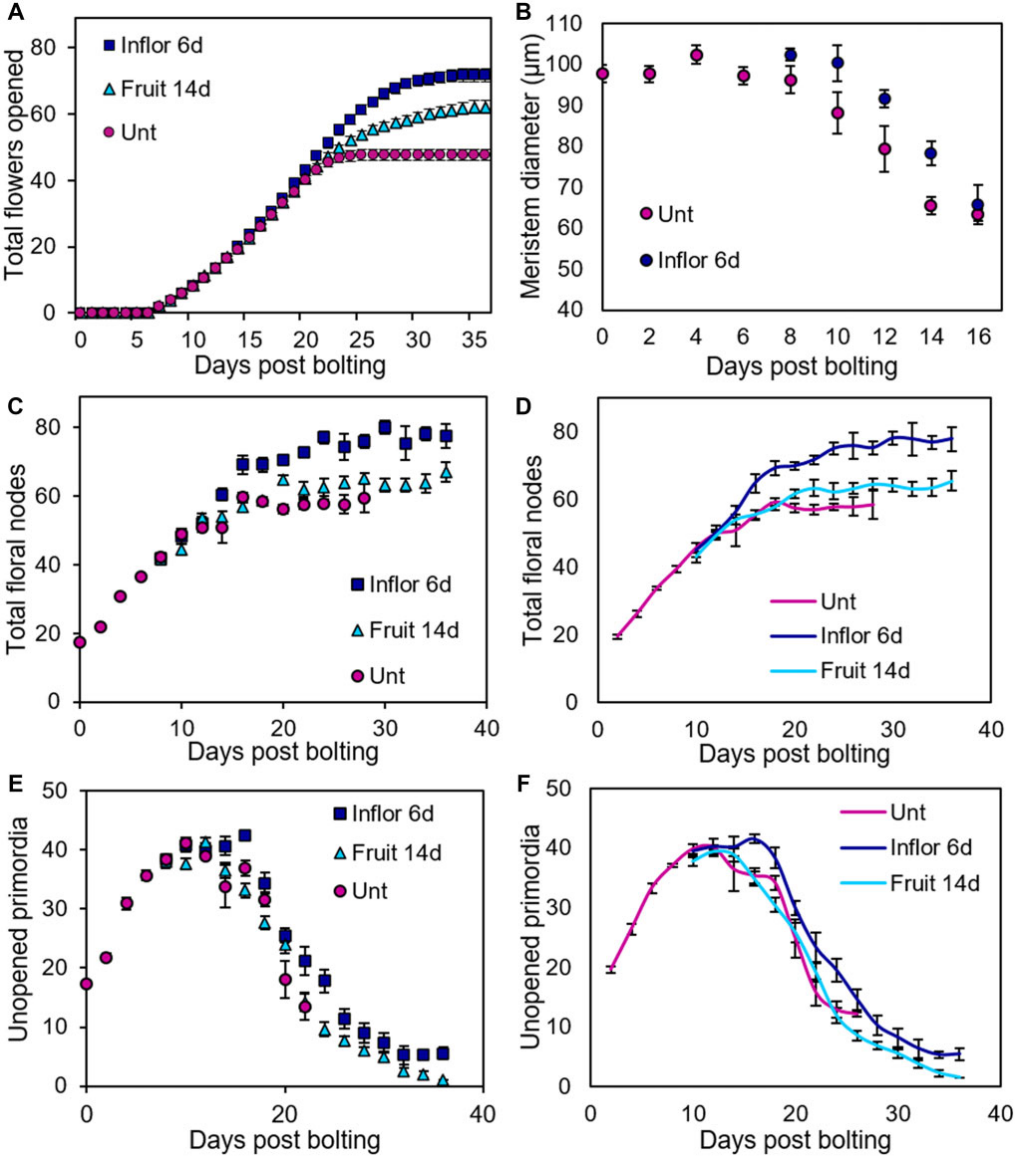
Systemic and local stimuli increase and extend flower opening. Based on observations in Figure 1, we performed four different treatments on flowering Col-0 plants. Firstly, we removed all inflorescences apart from the PI from the plant at 6 dpb (Inflor 6d). This time point was chosen as the earliest time point at which secondary inflorescences are visibly elongating. We also removed all fruits from the PI at 14 dpb and continuously thereafter (Fruit 14d). This time point was chosen as the earliest time point at which sufficient numbers of developed fruit are present for their removal to potentially make a difference. A, Scatter graph of cumulative flowers opened on the PI of each treatment each day post bolting; data collected nondestructively from individual plants. Error bars show sem, *n* = 10–13. B–F, A large population of Col-0 plants was grown under controlled conditions. The timing of visible bolting was recorded for each plant. Plants were randomly assigned to be sampled on a given number of days post bolting, and then destructively sampled at that time point. Time points were spaced every 2 days, and 3–12 plants were sampled for each time point. Error bars for all graphs show sem. B, Scatter graph showing mean IM diameter, from 0 to 16 dpb for control plants, and Days 6–16 from plants treated from Day 6 with inflorescence removal. C, Scatter graph showing the number of total floral nodes present from 0 to 28 dpb for control plants, and from Day 6 to 36/38 dpb for plants treated from Day 6 with inflorescence removal or Day 14 with fruit removal. D, Scatter graph showing the data from (C) plotted as a two-time point rolling average in order to show a slightly smoothed version of the data illustrating the overall trend. E, Scatter graph showing the total number of unopened floral primordia present in the inflorescence apex from 0 to 30 dpb for control plants, and from Day 6 to 36/38 dpb for plants treated from Day 6 with inflorescence removal or Day 14 with fruit removal. Buds and primordia were counted by dissecting buds from the bud cluster under a dissecting microscope. F, Scatter graph showing the data from (E) plotted as a two-time point rolling average in order to show a slightly smoothed version of the data illustrating the overall trend.

We first examined the rate of flower opening on the PI of Col-0 plants, which showed that both these treatments indeed increased the floral duration of the PI compared to untreated plants, which in this experiment again underwent inflorescence arrest at ∼24 dpb (Figure 5A). Removing inflorescences from 6 dpb resulted in an additional ∼25 flowers opening due to prolonged duration (by ∼8 days), rather than increased rate of opening (Figure 5A). Removing fruit from 14 dpb also resulted in prolonged duration (∼10 days), but with a slower rate of flower opening (approximately 15 additional flowers at ∼1.5/day; Figure 5A).

The qualitative differences between these treatments suggested that their effects arose from different developmental events. We therefore examined the timing of IM and inflorescence arrest in plants subjected to these treatments, using the same basic experimental design as in Figure 1. In this experiment, untreated plants underwent IM arrest at ∼18 dpb (Figure 5, C–F), and visible inflorescence arrest at ∼22 dpb (Figure 5C). Plants treated with inflorescence removal from 6 dpb showed a clear delay in IM arrest, continuing to initiate floral nodes for 5–6 days after control plants, and ultimately producing significantly more floral nodes (e.g. Day 26: *t* test, *P* < 0.05, *n* = 5; Figure 5, C and D). Consistent with this, plants also showed a delay in reduction of IM size between 8 and 16 dpb (Figure 5B). Intriguingly, these plants also showed a clear delay in floral arrest, even accounting for the delay in IM arrest; the plants continued to open new flowers for 10 days after IM arrest (until ∼32 dpb), and arrested with a bud cluster of only five primordia. Thus, compared to control plants, the treated plants flowered for 10 days longer, initiated an additional 15 flowers and opened an additional 25 (Figure 5, A–F).

In contrast, plants treated with local fruit removal after 14 dpb showed no clear alteration in the timing of IM arrest (Figure 5, C and D), but did have a small and statistically nonsignificant increase in floral node number (by ∼4 nodes; e.g. Day 26: *t* test, *P* > 0.05, *n* = 5; Figure 3, C and D). Conversely, they showed a very clear delay in floral arrest, continuing to open flowers for an additional ∼14 days until 36 dpb, resulting in the opening of an additional ∼15 flowers, at which point the bud cluster was essentially exhausted (Figure 5, E and F). The nature of inflorescence arrest was different in these plants compared to plants with global inflorescence removal, in the sense that they opened flowers until the bud cluster was essentially exhausted. Furthermore, in ∼30% of de-fruited plants, the IM was visibly consumed into a terminal flower or fruit (Supplemental Figure S1A), which was never seen in untreated plants, or those treated with global inflorescence removal, but was previously observed in *rock2* and *rock3* (Supplemental Figure S1B).

Thus, global inflorescence removal and local fruit removal both delay inflorescence arrest, but do so in qualitatively (and quantitatively) different ways. Early global inflorescence removal delayed both IM and floral arrest, but plants eventually underwent a “normal” inflorescence arrest. Conversely, local fruit removal only delayed floral arrest, with no obvious change in IM activity (Figure 5D), and led to an inflorescence arrest by extinction, with some terminal flower formation. These data therefore show that the two stages of inflorescence arrest are functionally distinct, and respond to different internal stimuli. Floral arrest is a highly sensitive process, which can be delayed by local deficits in reproductive success, whereas IM arrest is only sensitive to large deficits in reproductive success at the level of the whole plant. The separability of the two processes therefore likely gives plants two distinct strategies to flexibly respond to changes in their own reproductive success.

### Global inflorescence and local fruit removal can reactivate IM and FM activity

**Figure 6:**
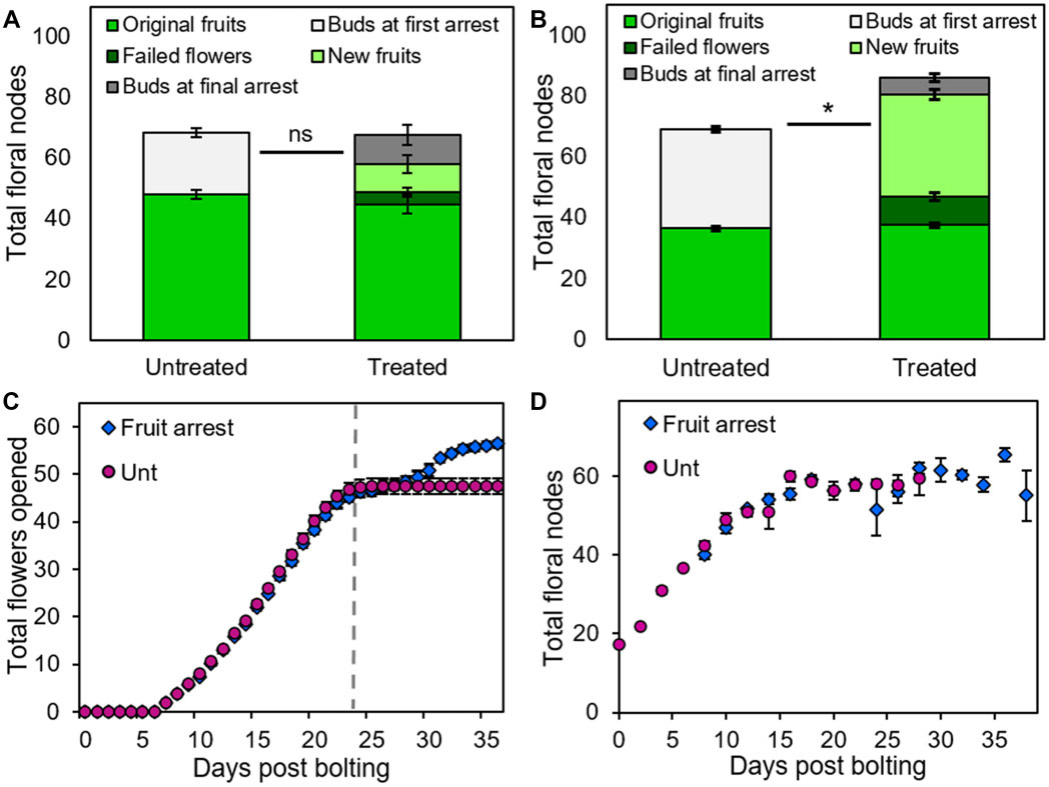
Reactivation of flower opening by inflorescence removal. A, Stacked bar graph, showing the number of floral nodes on the PI produced in Col-0 plants either left untreated or treated by removal of all other inflorescences after arrest of the PI. The total floral nodes (i.e. the height of the full stack) are broken down into fruit produced on the PI during initial flowering (mid-green, lower bars), plus either (a) the number of buds and primordia remaining in the bud cluster at first arrest (light gray; untreated plants only) or (b) the number of failed flowers (dark green), new fertile fruits (light green), and the number of buds and primordia remaining in the bud cluster at final arrest (dark gray; treated plants only). Error bars indicate sem, *n* = 10 (untreated), 6 (treated). ns = no significant difference (*t* test, *P* >0.05) between total floral node number between treated plants and untreated controls. B, Stacked bar graph, showing the number of floral nodes on the PI produced in L*er* plants either left untreated or treated by the removal of all other inflorescences after the arrest of the PI. The total floral nodes (i.e. the height of the full stack) are broken down into fruit produced on the PI during initial flowering (mid-green, lower bars), plus either (a) the number of buds and primordia remaining in the bud cluster at first arrest (light gray; untreated plants only) or (b) the number of failed flowers (dark green), new fertile fruits (light green), and the number of buds and primordia remaining in the bud cluster at final arrest (dark gray; treated plants only). Error bars indicate sem, 11 (untreated), 9 (treated). Asterisk indicates significant difference (*t* test, *P*<0.0001) between total floral node number between treated plants and untreated controls. C, Scatter graph of cumulative flowers opened on the PI of each treatment. Data were collected nondestructively from 11 individual plants per treatment, assessed daily post bolting. “Fruit arrest” plants were treated from 1 day after their arrest by the removal of all fruit on the PI, then left to respond; “Unt” plants were left untreated. The point of treatment for fruit arrest plants has been normalized to 24-day post anthesis (dashed line), such that Day 25 shows plants 1-day post-treatment, etc. Error bars show sem. D, Scatter graph of total floral nodes present on the PI of each treatment. A large population of Col-0 plants was grown under controlled conditions; “Fruit arrest” plants were treated from 1 day after PI arrest by the removal of all fruit on the PI, then left to respond. “Unt” plants were left untreated. The timing of visible bolting was recorded for each plant; plants were then randomly assigned to be sampled on a given number of days post bolting (or post-arrest), and then destructively sampled at that time point. Time points were spaced every 2 days, and five to seven plants were sampled for each time point. Error bars for all graphs show standard error of the mean. The point of treatment for fruit arrest plants has been normalized to 24 dpb, such that Day 25 shows plants 1-day post-treatment, etc. Error bars show sem.

Hensel et al. (1994) showed that individual inflorescences can also be induced to reactivate after inflorescence arrest in response to inflorescence or fruit removal. Given our data, we questioned whether this occurs by reactivation of IM activity, flower opening, or both. We therefore treated Col-0 plants with global inflorescence removal after arrest of the PI, which promoted re-activation after an ∼8-day delay, beginning with ∼4 of the characteristic “failed” flowers (Figure 2C), before successful opening of ∼9 new fertile flowers (Figure 6A). Treated plants did not produce any additional floral nodes in total (Figure 6A;*t* test, *P* > 0.05, *n* = 6–10), showing these changes are achieved by re-activation of flower opening without new IM activity. We also found that local fruit removal after arrest in Col-0 was able to trigger the same level of re-activation of flower opening, although the process occurred more quickly (within ∼4 days; Figure 6C). Again, this occurs without any significant increase in the number of floral nodes initiated between treated and untreated plants (e.g. Day 28, Mann–Whitney *U* test, *P* > 0.05, *n* = 3–6; Figure 6D).

We also tested global inflorescence removal after arrest of the PI in the L*er* ecotype, in which Hensel et al. (1994) performed their experiments. In contrast to Col-0, we found that inflorescence re-activation in L*er* involved reactivation of both IM activity and flower opening, with treated plants opening an additional ∼33 new fertile flowers (and nine failed flowers), but also showing a clear increase of ∼17 total floral nodes over untreated plants (*t* test, *P* < 0.0001, *n* = 9–11; Figure 6B). This unexpected ecotypic difference in IM reactivation potential between Col-0 and L*er* is intriguing, and might reflect the known roles of ERECTA in meristem maintenance (Mandel et al., 2014, 2016; Zhang et al., 2021); it is possible that it is the *erecta* mutation itself that contributes to the difference between the ecotypes. Irrespectively, these data again emphasize the separability of IM and floral arrest as developmental processes.

### Cytokinin signaling is needed for homeostatic regulation of inflorescence arrest

Our data reveal some clear similarities between *rock2* and *rock3* mutants, and treatments that affect whole-plant reproductive success. The phenotype of *rock3* mutants is very similar to the effect of local fruit removal, with a delay in floral arrest, including the formation of terminal flowers in ∼30% of IMs (Supplemental Figure S1B). The phenotype of *rock2*, on the other hand, is similar to the effect of global inflorescence removal, with a delay in both IM arrest and floral arrest. However, *rock2* mutants also showed terminal flowers in ∼30% of IMs, which was never seen in plants treated with global inflorescence removal. Thus, the phenotype of *rock2* seems analogous to the effects of global inflorescence removal and local fruit removal.

**Figure 7:**
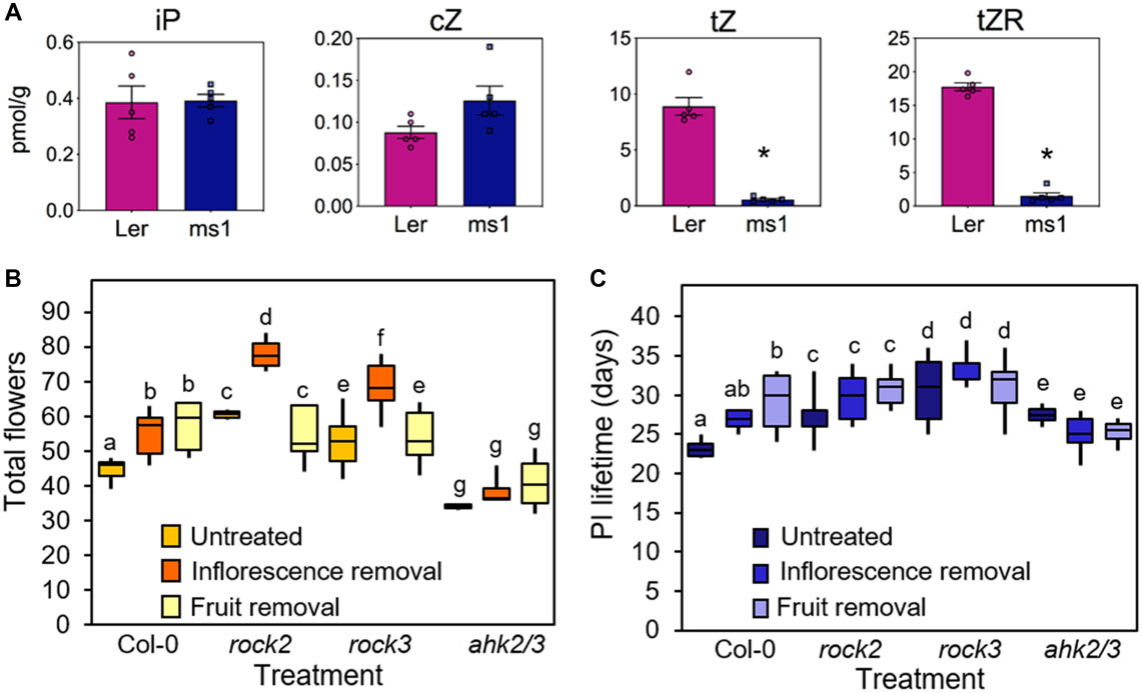
Cytokinin signaling is needed for homeostatic regulation of inflorescence arrest. A, Concentration (pmol/g fresh weight) of the free cytokinin bases isopentenyladenine (iP), cis-Zeatin (cZ), trans-Zeatin (tZ; biologically active cytokinins), and trans-Zeatin riboside (tZR; major root-to-shoot transport form) in the fertile or sterile fruit of L*er* and *ms1* plants. *n* = 5 biologically independent samples (shown by overlying circles), error bars show sem. Asterisk indicates significant difference from L*er* control (Mann–Whitney *U* test, *P* <0.05, *n* = 5). B, Box plots showing the total number of opened flowers on the PI of Col-0, *ahk2/3*, *rock2*, *rock3*, either untreated (yellow boxes), or treated with inflorescence removal at 9 dpb (orange boxes), or fruit removal at 14 dpb (beige boxes). Boxes indicate the interquartile range and internal line shows the median. Whiskers indicate maximum and minimum values. Bars with the same letter are not statistically different from each other (ANOVA + Tukey HSD, calculated separately within each genotype, *n* = 2–9). C, Box plots showing the inflorescence lifetime in days of the PI of Col-0, *ahk2/3*, *rock2*, *rock3*, either untreated (dark yellow boxes), or treated with inflorescence removal at 9 dpb (orange boxes), or fruit removal at 14 dpb (light yellow boxes). Boxes indicate the interquartile range and internal line shows the median. Whiskers indicate maximum and minimum values. Bars with the same letter are not statistically different from each other, (ANOVA + Tukey HSD, *P*<0.05, calculated separately within each genotype, *n* = 2–9). Data representative of multiple independent experiments.

Overall, our data suggest a model in which inflorescences and fruits act as sinks for cytokinin, and that this effect governs the timing of IM arrest and floral arrest. In support of this idea, we found that WT fertile fruit have much higher levels of trans*-*Zeatin riboside (*t*ZR; the main transport form of trans*-*Zeatin (*t*Z); Hirose et al., 2008), and the signaling-active *t*Z form itself, compared to sterile fruit of the *male sterile1* mutant (Figure 7A). Conversely, sterile and fertile fruit contained similar quantities of isopentenyladenine (iP) and cis-Zeatin (*c*Z) cytokinins, showing there is not a general reduction in cytokinin in sterile fruit (Figure 7A). We thus hypothesize that, as new inflorescences and fruits initiate during flowering, there is a resultant progressive dilution of cytokinin across the shoot system, which leads to reduced cytokinin levels in the IMs (Figure 3, A–H). This reduction in cytokinin contributes to inflorescence arrest by decreasing IM activity and flower opening (Figure 4). Conversely, if cytokinin sinks are removed (Figure 5) or if cytokinin sensitivity is increased (Figure 4), IM arrest and floral arrest are delayed. Consistent with this model, we found that plants treated with inflorescence removal at 6 dpb showed a dramatic increase in cytokinin signaling in the IM at 12 dpb consistent with the prolonged activity of these IMs, before returning to pre-treatment levels by 15 dpb (Figure 3, A–H).

As a critical test of this model, we therefore hypothesized that *ahk2/3* should fail to respond to either inflorescence or fruit removal and that conversely *rock2* and *rock3* should over respond to inflorescence removal—but not to fruit removal, since they already open almost all flowers they produce. To test these hypotheses, we performed inflorescence removal at 9 dpb and fruit removal at 14dpb treatments in *ahk2/3*, *rock2*, and *rock3* mutants. Consistent with our hypothesis, we found that *ahk2/3* showed very little response to either treatment and no statistically significant difference in either the number of flowers opened or the overall lifetime of the PI (Figure 7, B and C). Similarly, we saw no significant difference in flowers opened or PI lifetime in *rock2* and *rock3* in response to fruit removal (Figure 7, B and C). However, we saw an increase in the number of flowers opened in both *rock2* and *rock3* compared to Col-0, thus strongly supporting our hypothesis (Figure 7B).

## Discussion

### Inflorescence arrest in Arabidopsis is a complex developmental phenomenon

Previous work has tended to view inflorescence arrest in Arabidopsis as a process driven by changes in the activity of the IM (Hensel et al., 1994; Wuest et al., 2016; Balanzà et al., 2018; Merelo et al., 2022). However, the fact that Arabidopsis inflorescences arrest with a cluster of unopen flowers calls into question this idea. If IM arrest directly led to inflorescence arrest, then inflorescence arrest should occur because of a lack of new flowers to open (as is indeed the case in many species). The results presented here clearly demonstrate that inflorescence arrest in Arabidopsis usually involves the arrest of both IMs and developing flowers, and show that the timing of inflorescence arrest is more directly determined by the timing of the floral arrest, rather than IM arrest. Our results show that IM and floral arrest are separate and separably regulated processes, which can be delayed in response to global inflorescence and local fruit removal. A surprising aspect of our results is just how early the events that lead to inflorescence arrest occur. IM arrest occurs 5–9 days before visible inflorescence arrest, and the last flower to fully mature is typically initiated just after anthesis, ∼5 days *before* IM arrest (Figure 1). Floral arrest seems to occur shortly after IM arrest, suggesting that both processes might occur in response to the same external stimuli, which are present during this phase in flowering.

Our results also provide important information about the reversibility of arrest. Consistent with earlier reports, we show that IMs in L*er* enter a quiescent state upon arrest (Wuest et al., 2016), from which they can be reactivated in response to a loss of reproductive structures globally (Hensel et al., 1994; Merelo et al., 2022). However, we find that although Col-0 IM activity can be extended by global loss of reproductive structures (Figure 2), Col-0 IMs cannot be reactivated after IM arrest (Figure 3). The reason for this difference between Col-0 and L*er* ecotypes is unclear, but it represents a promising avenue for future investigation.

### Auxin and floral arrest

While the process of IM arrest has previously been described (Wang et al., 2020; Merelo et al., 2022), our results define the process of floral arrest. Remarkably, we show that floral arrest only affects flowers at Stage 9 or below, and has the effect of halting them in their current developmental stage. We also show that floral arrest is reversible in response to both global and local loss of reproductive structures in both Col-0 and L*er*. We show that all unopened flowers can re-initiate development after arrest, but those that are initially arrested between Stages 6–9 cannot develop further than the ‘checkpoint’ at Stage 9. Flowers that arrested at Stage 5 and younger can, however, fully develop and go on to produce fertile fruit.

As we discussed above, the Stage 5 checkpoint likely relates to the fact that the FM is still active in flowers younger than Stage 5. These flowers can thus probably reactivate at the level of FM activity and complete their development. But what about the Stage 9 checkpoint? One possibility is that this checkpoint relates to organ and stem elongation. Stage 9, following Smyth et al. (1990), is the stage at which the floral organs begin to rapidly elongate toward their full sizes. It is notable that there is very little internode elongation between the flowers in the arrested bud cluster (Figure 1E), and that this remains the case even when there is reactivation of the inflorescence (Figure 2C). In general, there is a marked reduction in internode elongation toward the end of inflorescence development (Goetz et al., 2021), culminating in the arrested bud-cluster. It thus seems that floral arrest takes place in an environment of extremely reduced stem/organ elongation, and indeed, as a developmental process, might largely constitute the absence of such elongation.

This becomes particularly interesting in light of our previous study, which showed that later-produced fruit (i.e. those formed in the later 50% of the period between anthesis and inflorescence arrest) are locally required for inflorescence arrest, and that auxin export from these late fruit is key to this effect (Ware et al., 2020). In this study, our developmental analysis clarifies that this auxin-related mechanism likely specifically relates to floral arrest and not IM arrest, given that we show here that local fruit removal does not substantially affect IM activity (Figure 5). Furthermore, by the point in flowering that these late fruits begin to form, the IM has usually already arrested. We previously proposed that auxin exported by fruit acts by preventing the export of auxin from other organs via a canalization-dependent mechanism (Ware et al., 2020). It is also notable that auxin transport in the inflorescence diminishes toward the end of inflorescence development, and that auxin appears to build up in the inflorescence stem (Goetz et al., 2021). This therefore suggests a model in which the cumulative auxin exported by fruit triggers floral arrest by preventing the further export of auxin from flowers at Stage 9 or younger, which in turn prevents both organ elongation in the flower and elongation in the adjacent internode. Flowers above Stage 10 presumably already have a well-established, canalized auxin export into the stem, and so are immune to this effect (Figure 2), while flowers at Stage 5 or below with an active FM can re-establish auxin export if nearby fruit are removed. However, those between Stages 6 and 9 are unable to re-form a link and therefore fail to fully develop. This model therefore provides a testable hypothesis for the nature and regulation of floral arrest for future studies.

### The role of cytokinin in inflorescence arrest

Our results clearly demonstrate that cytokinin is an important regulator of two-stage inflorescence arrest in Arabidopsis. Our results show that there is a clear decline in cytokinin signaling in the IM in the lead-up to IM arrest, that cytokinin treatment can delay IM and floral arrest, and that cytokinin mutants show strong perturbations in the progression of inflorescence lifetime, including an inability of *ahk2 ahk3* mutants to respond to inflorescence or fruit removal (Figures 5 and 6). Our results are consistent with the recent publication of Merelo et al. (2022), who also demonstrated that cytokinin signaling diminishes over IM lifetime, and that cytokinin treatment can delay IM arrest. However, our results provide additional information relative to those of Merelo et al. (2022), by (1) providing clear genetic evidence for the role of cytokinins in IM arrest, (2) demonstrating the role of cytokinins in floral arrest as well as IM arrest, and (3) placing the role of cytokinin within a clear developmental framework, namely the re-distribution of reproductive effort based on reproductive success elsewhere on the plant.

In particular, we show that the *rock2* and *rock3* mutants, previously implicated in inflorescence arrest (Bartrina et al., 2017), differentially regulate IM and floral arrest, consistent with the high expression of *AHK2* in IMs and FMs, and of *AHK3* in FMs. Remarkably, our results show that *rock2* and *rock3* phenotypes closely resemble the effect of global inflorescence and local fruit removal respectively, implicating cytokinin in the coordination of arrest events across the plant in response to systemic and local reproductive success. Consistent with this, we show that the cognate loss-of-function *ahk2 ahk3* mutants are unable to respond to inflorescence or fruit removal by extending the duration of IM or flower opening activity.

We propose that both inflorescences and fertile fruit might act as sinks for *t*Z cytokinin from the root system (Figure 7A) and that the continued production of these new cytokinin sinks during flowering causes a progressive dilution in root-derived *t*Z availability within the shoot. This reduces cytokinin levels in any given inflorescence, which leads to a reduction in IM size and ultimately IM arrest, followed shortly after by floral arrest. This hypothesized re-distribution of *t*Z cytokinin between sinks in the shoot would present an elegantly simple system for plants to adjust inflorescence lifetime to compensate for reduced reproductive success. In particular, it can be seen that a local failure of external pollination—not a factor in highly self-fertile Arabidopsis, but a key consideration in most other Brassicaceae—would trigger the compensatory maturation of additional flowers by preventing cytokinin sinks/auxin sources from developing. A more dramatic loss of inflorescences by e.g. herbivory would trigger both the development of additional inflorescences (Walker et al., 2021) and prolong the lifetime of existing inflorescences.

## Materials and methods

### Plant growth conditions

Plants for phenotypic and microsurgical experiments were grown on John Innes compost under a standard 16-h/8-h light/dark cycle (20°C) in either controlled environment rooms with light provided by fluorescent tubes at a light intensity of ∼120 µmol·m^−2^·s^−1^ or in glasshouses with supplemental lighting. Plants for cytokinin application experiments were grown on John Innes No. 3 compost under the same light/dark cycle but at 22°C/18°C, with light provided by fluorescent tubes at an intensity of ∼150 µmol·m^−2^·s^−1^.

### Plant materials

Arabidopsis (*Arabidopsis thaliana*) WTs Col-0 and L*er* were used as indicated. The following lines are all in a Col-0 background and have previously been described; *TCSn:GFP* (Liu and Müller, 2017); *rock2*, *rock3* (Bartrina et al., 2017); *ipt3-2 ipt5-2 ipt7-1* (Miyawaki et al., 2006); *ahk2-2 ahk3-3* (Higuchi et al., 2004).

### Flowering assessments and meristem measurements

To define the manner in which Arabidopsis inflorescences arrest, we grew a large population of WT Col-0 Arabidopsis under long-day conditions. Each plant was pre-allocated to be sampled at a given time point after its primary shoot axis had “bolted”. In this way, approximately six plants were sampled for each time point, with the time points being at 1-day intervals post bolting. Sampling was destructive, so we could not just measure the same plants each day post bolting. For each plant, we recorded (1) the number of open and previously opened flowers; (2) the number of as-yet-unopened floral buds including all floral primordia visible by dissecting the inflorescence apex under a microscope (Figure 1F); and (3) the cumulative number of floral nodes initiated by each inflorescence at that time point (i.e. the sum of 1 and 2).

Genotypes (where relevant) and age of collection were randomized across trays, and the date of bolting was recorded for each plant. When ready for collection, the entire bud cluster above the uppermost open flower (where present) was removed from the plant with forceps. In the event of collection prior to anthesis, the entire bud cluster was collected. All open flowers on the PI were counted prior to collection. The apex of the inflorescence (containing all unopened flowers) was removed from each plant and mounted into a plate containing solidified water agarose to prevent desiccation, with the meristem facing upward. These were then dissected under a dissecting microscope using forceps and a micro-scalpel. The total number of unopened flowers and floral primordia were counted, with as many as possible being removed. The dissected apices were imaged under a Keyence VHX-7000 digital microscope, using a VH-Z100R RZx100-x1000 real zoom lens. Images were loaded into ImageJ (Schneider et al., 2012), where the mean of three meristem diameters was calculated using methodology adapted from Landrein et al. (2015).

### Micro-surgical experiments

Inflorescence removal as described in Figures 2 and 4 was carried out by removing all inflorescences except the PI with scissors at 6 dpb. Plants were then monitored every subsequent 2–3 days and newly developed branches were removed until sample collection. Fruit removal treatments were carried out at either 14 dpb or on the day of final flower opening as indicated. All developed fruits and open flowers were removed from the PI using forceps. Plants were monitored every 1–3 days, with all additional flowers being removed until sample collection.

### Confocal imaging

Inflorescence apices of *TCSn:GFP* plants were prepared, mounted, and dissected as described above. The agar plates were then flooded with distilled water to allow water-dipping lenses to be used to image the meristem. Meristems were imaged using a Zeiss LSM880 with a 20× water dipping lens. Excitation was performed using 488 nm (10% laser power) and 555 nm (5%) lasers. Chloroplast autofluorescence was detected above 600 nm, and green fluorescent protein fluorescence below 555 nm. *Z*-stacks were taken of each meristem, covering the whole depth of the meristem dome, and then a maximum intensity projection was made of the *z*-stack. Quantification was performed on these projections using ImageJ (Schneider et al., 2012). The same microscope settings were used for all meristems.

### RT–qPCR

Col-0 plants were grown and their date of anthesis was recorded. Inflorescence apices (including all unopened buds) of the PI (four to eight individual plants pooled per biological replicate) were subsequently harvested, snap frozen, and stored at −80°C until RNA extraction. RNA was extracted from samples using a QIAGEN RNeasy plant mini kit as per the manufacturer’s instructions (including DNAse treatment). cDNA was synthesized using Superscript IV reverse transcriptase with 1 μg of input RNA per sample. RT–qPCR was performed on an Analytik-Jena qTOWER using PowerUp SYBR Green mastermix (Thermo-Fisher), with 10-µL reactions containing 0.25 µL forward and reverse primers (100-µM stock), 5-µL SYBR green mastermix, 1-µL cDNA, and 3.5 µL water. Cp values were calculated using the manufacturer’s software and subsequently compared via the 2^–ΔΔCt^ method, normalized to the average 0 dpb values, with the housekeeping gene PP2A3 as an internal control. The results presented are the average of four biological replicates with three technical replicates each.

Primers: ARR5-F: tcagagaacatcttgcctcgt; ARR5-R: atttcacaggcttcaataagaaat; ARR7-F: ccggtggagatttgactgtt; ARR7-R: tccactctctacagtcgtcacttt; PP2A3-F: tccgtgaagctgctgcaaac; PP2A3-R: caccaagcatggccgtatca.

### Cytokinin applications

Cytokinin applications were performed via application in lanolin to emerged fruits of L*er* plants using a micropipette tip, the same methodology as in Ware et al. (2020). Either 10 µL (1 mg·g^−1^ treatment) or 1 µL (0.1 mg·g^−1^ treatment) of 100 mg·g^−1^ 6-benzlyaminopurine stock in DMSO was added to lanolin with 1-µL dye to ensure even incorporation or DMSO and dye alone for the corresponding mock treatments. Treatments were performed at the same points as measurements, and the treatment regimen was initiated 12 days after anthesis of the first flower.

### Cytokinin measurements

For the cytokinin analysis of the fertile or sterile fruit of L*er* and *ms1* plants, ∼10 mg of fresh-weight material was used per sample (*n* = 5). Samples were extracted in modified Bieleski buffer (methanol/water/formic acid, 15/4/1 [v/v/v]) with a mixture of stable isotopically labeled internal standards added to each sample for precise quantification (Hoyerová et al., 2006). The purification of isoprenoid cytokinins (CKs) was carried out according to Dobrev and Kamínek (2002) using the MCX column (30 mg of C18/SCX combined sorbent with cation-exchange properties). Analytes were eluted by two-step elution using a 0.35-M NH_4_OH aqueous solution and 0.35-M NH_4_OH in a 60% MeOH (v/v) solution. Samples were afterward evaporated to dryness under vacuum at 37°C. Prior to analysis, the samples were dissolved in 40-µL 10% MeOH (v/v). Mass spectrometric analysis and quantification were performed using an ultra-high-performance liquid chromatography–tandem mass spectrometry system consisting of a 1290 Infinity Binary LC System coupled to a 6490 Triple Quad liquid chromatography/mass spectrometry (LC/MS) System with Jet Stream and Dual Ion Funnel technologies (Agilent Technologies, Santa Clara, CA, USA). Ultra-high-performance liquid chromatography–electrospray ionization–tandem mass spectrometry method parameters were adapted from (Svačinová et al., 2012).

### Experimental design and statistics

The sample size for each experiment is described in the figure legends. For plant growth experiments, each sample was a distinct plant. For cytokinin measurements, each sample was a set of tissue pooled from multiple plants; each sample was distinct. For data analysis, we tested data for normality to determine the most appropriate statistical test, except when mixed-effects models were used, where instead sphericity was not assumed and the Greenhouse–Geisser correction was applied. The statistical tests performed for each experiment are described in the text and/or in the figure legends. For Sidak’s multiple comparisons, individual variances were calculated for each comparison.

## Supplementary Material

kiac514_Supplementary_Data

## Data Availability

All figures in this manuscript are associated with raw data. All raw data will be made available upon request to the corresponding author. Sequence data from this article can be found in the GenBank/EMBL data libraries under the following accession numbers: AHK2: At5g35750; AHK3: At1g27320; ARR5: At3g48100; ARR7: At1g19050.

## Supplemental data

The following materials are available in the online version of this article.

**Supplemental Figure S1.** Systemic and local stimuli increase and extend flower opening.

**Supplemental Figure S2.** Cytokinin signaling regulates IM and floral arrest.

## Notes

C.H.W., A.W., and T.B. performed experiments and analyzed the data. J.S. and K.L. carried out the cytokinin analysis. C.H.W., T.B., and Z.W. designed the study. All authors contributed to writing the manuscript.

The author responsible for distribution of materials integral to the findings presented in this article in accordance with the policy described in the Instructions for Authors (https://academic.oup.com/plphys/pages/general-instructions) is: Tom Bennett (t.a.bennett@leeds.ac.uk).
