# Supplementary material for: Cytokinin signaling regulates two-stage inflorescence arrest in Arabidopsis
Source: Plant Physiol. 2022 Nov 4;191(1):479–95. doi: 10.1093/plphys/kiac514 (PMC9806609; doi:10.1093/plphys/kiac514)
Supplement: kiac514_Supplementary_Data [file kiac514_supplementary_data.pdf]

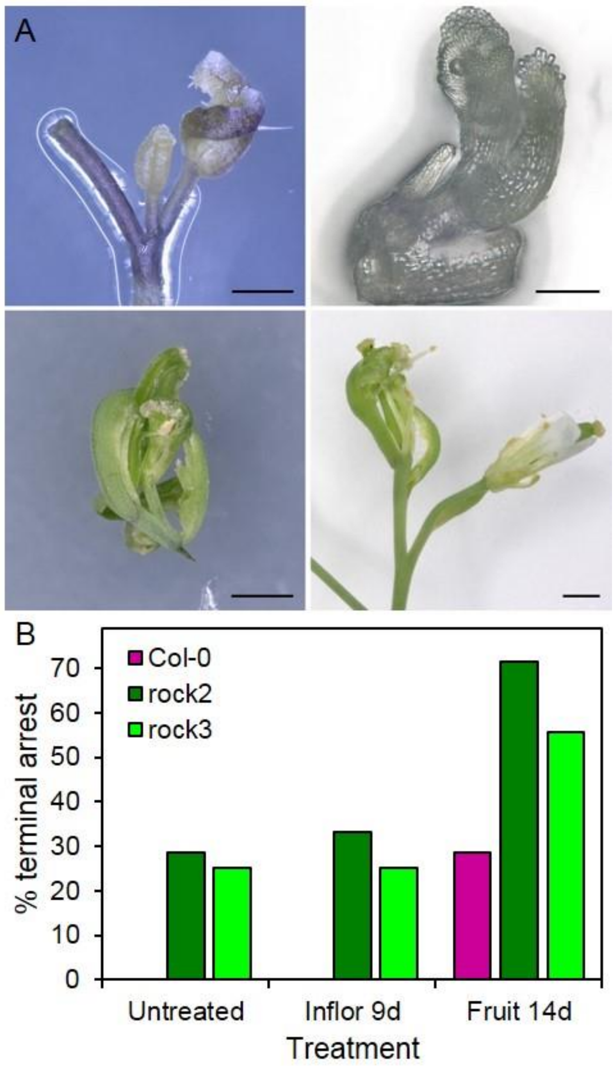

Supplemental Figure S1: **Systemic and local stimuli increase and extend flower opening**

A) Light micrographs showing terminal flowers/fruit resulting from terminal differentiation of the IM in plants treated with fruit removal at 14 days post bolting (dpb) and continuously thereafter. Bar indicates 500µm (top left), 100µm (top right), or 1000µm (bottom left and right).

(B) Bar graph showing frequency of terminal flower/fruit occurrence in Col-0, *rock2* and *rock3* plants either untreated, or treated by removal of all inflorescences apart from the primary inflorescence (PI) at 9dpb (Inflor 9d) or removal of all fruits from the PI at 14dpb and continuously thereafter (Fruit 14d). n = 6-9 plants per genotype/treatment.

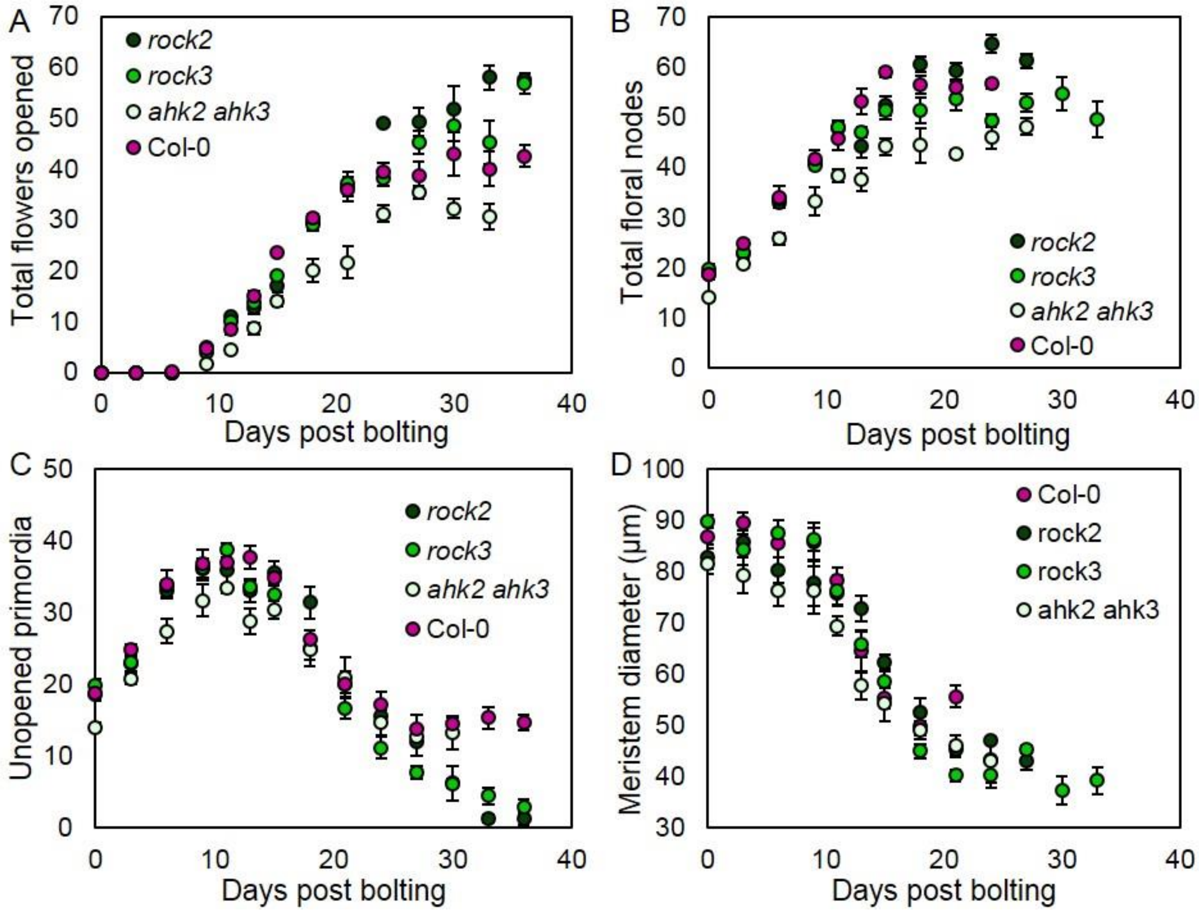

Supplemental Figure S2: Cytokinin signalling regulates IM and floral arrest

(**A-D**) Large populations of Col-0, *ahk2/3*, *rock2* and *rock3* plants were grown under controlled conditions. The timing of visible bolting was recorded for each plant. Plants were randomly assigned to be sampled on a given number of days post-bolting, and then destructively sampled at that timepoint. Timepoints were spaced every 2-3 days, and 3-12 plants sampled for each timepoint. Error bars for all graphs show standard error of the mean. These are the raw data, which are re-drawn in Figures 5A-D as two-timepoint rolling averages of this data. (**A**) Scatter graph showing mean opened flowers, at each timepoint from 0-33/36dpb for each genotype. (**B**) Scatter graph showing the number of total floral nodes present at each timepoint from 0-24/27/36dpb for each genotype. (**C**) Scatter graph showing the number of unopened primordia (floral buds and primordia) present in the inflorescence apex at each timepoint from 0-30/36dpb for each genotype. **D**) Scatter graph showing mean IM diameter at each timepoint from 0-24dpb for each genotype.
